# Evaluating the performance of a clinical genome sequencing program for diagnosis of rare genetic disease, seen through the lens of craniosynostosis

**DOI:** 10.1038/s41436-021-01297-5

**Published:** 2021-08-25

**Authors:** Zerin Hyder, Eduardo Calpena, Yang Pei, Rebecca S. Tooze, Helen Brittain, Stephen R. F. Twigg, Deirdre Cilliers, Jenny E. V. Morton, Emma McCann, Astrid Weber, Louise C. Wilson, Andrew G. L. Douglas, Ruth McGowan, Anna Need, Andrew Bond, Ana Lisa Taylor Tavares, Ellen R. A. Thomas, Genomics England Research Consortium, John C. Ambrose, Prabhu Arumugam, Roel Bevers, Marta Bleda, Christopher R. Boustred, Georgia C. Chan, Greg Elgar, Tom Fowler, Adam Giess, Angela Hamblin, Shirley Henderson, Tim J. P. Hubbard, Rob Jackson, Louise J. Jones, Dalia Kasperaviciute, Melis Kayikci, Athanasios Kousathanas, Lea Lahnstein, Sarah E. A. Leigh, Ivonne U. S. Leong, Javier F. Lopez, Fiona Maleady-Crowe, Meriel McEntagart, Federico Minneci, Loukas Moutsianas, Michael Mueller, Nirupa Murugaesu, Peter O’Donovan, Chris A. Odhams, Christine Patch, Mariana Buongermino Pereira, Daniel Perez-Gil, John Pullinger, Tahrima Rahim, Augusto Rendon, Tim Rogers, Kevin Savage, Kushmita Sawant, Afshan Siddiq, Alexander Sieghart, Samuel C. Smith, Alona Sosinsky, Alexander Stuckey, Mélanie Tanguy, Simon R. Thompson, Arianna Tucci, Matthew J. Welland, Eleanor Williams, Katarzyna Witkowska, Suzanne M. Wood, Susan L. Hill, Zandra C. Deans, Freya Boardman-Pretty, Mark Caulfield, Richard H. Scott, Andrew O. M. Wilkie

**Affiliations:** 1 Genomics England, London, UK; 2 Manchester Centre for Genomic Medicine, Manchester University NHS Foundation Trust, Manchester, Greater Manchester UK; 3 Clinical Genetics Group, MRC Weatherall Institute of Molecular Medicine, University of Oxford, Oxford, UK; 4 West Midlands Regional Clinical Genetics Service and Birmingham Health Partners, Birmingham Women’s and Children’s Hospitals NHS Foundation Trust, Birmingham, UK; 5 Oxford Centre for Genomic Medicine, Oxford University Hospitals NHS Foundation Trust, Oxford, UK; 6 Department of Clinical Genetics, Liverpool Women’s NHS Foundation Trust, Liverpool, UK; 7 Clinical Genetics Service, Great Ormond Street Hospital, London, UK; 8 Wessex Clinical Genetics Service, University Hospital Southampton NHS Foundation Trust, Southampton, UK; 9 Human Development and Health, Faculty of Medicine, University of Southampton, Southampton, UK; 10 West of Scotland Centre for Genomic Medicine, Queen Elizabeth University Hospital, Glasgow, UK; 11 South East Regional Genetics Service, Guy’s and St Thomas’ NHS Trust, London, UK; 12 Genomics Unit, NHS England & NHS Improvement, London, UK; 13 William Harvey Research Institute, Queen Mary University of London, London, UK

## Abstract

**Purpose:**

Genome sequencing (GS) for diagnosis of rare genetic disease is being introduced into the clinic, but the complexity of the data poses challenges for developing pipelines with high diagnostic sensitivity. We evaluated the performance of the Genomics England 100,000 Genomes Project (100kGP) panel-based pipelines, using craniosynostosis as a test disease.

**Methods:**

GS data from 114 probands with craniosynostosis and their relatives (314 samples), negative on routine genetic testing, were scrutinized by a specialized research team, and diagnoses compared with those made by 100kGP.

**Results:**

Sixteen likely pathogenic/pathogenic variants were identified by 100kGP. Eighteen additional likely pathogenic/pathogenic variants were identified by the research team, indicating that for craniosynostosis, 100kGP panels had a diagnostic sensitivity of only 47%. Measures that could have augmented diagnoses were improved calling of existing panel genes (+18% sensitivity), review of updated panels (+12%), comprehensive analysis of de novo small variants (+29%), and copy-number/structural variants (+9%). Recent NHS England recommendations that partially incorporate these measures should achieve 85% overall sensitivity (+38%).

**Conclusion:**

GS identified likely pathogenic/pathogenic variants in 29.8% of previously undiagnosed patients with craniosynostosis. This demonstrates the value of research analysis and the importance of continually improving algorithms to maximize the potential of clinical GS.

## INTRODUCTION

Early evaluations of genome sequencing (GS) of rare disorders in a research setting showed that it could provide diagnostic enhancement of 21–42%, according to clinical context [1–3]. This has led to initiatives to introduce GS into clinical diagnostics. In the UK, the 100,000 Genomes Project (100kGP), delivered by National Health Service (NHS) England through 16 NHS Genomic Medicine Centres (GMCs) together with Genomics England (GE), was inspired by the potential for GS to provide patient benefit in the NHS, offering prompter diagnoses and improving prediction and prevention [4–6]. Genome sequencing is particularly valuable in conditions presenting with variable phenotypes or nonspecific clinical features, where the number of contributory genes may be extensive, and can identify noncoding variants and unravel new pathogeneses of disease [7, 8].

Recruitment of participants into the 100kGP was carried out by GMCs between 2015 and 2018; in the rare disease program, GS has been performed on 71,597 participants in 36,012 families. An automated pipeline, centered on the use of updateable, crowd-sourced and disease-focused panels (PanelApp) [9] was created by GE for processing, calling, and prioritizing genome sequence variants, and the results were returned to the recruiting GMC to evaluate and potentially validate [4]. The rate of diagnoses achieved by the GE/GMC pipeline for rare diseases is currently 20.3%.

The 100kGP allowed access to de-identified clinical and genomic data in the Research Environment to academic researchers accredited as members of one of 49 GE Clinical Interpretation Partnerships, investigating a wide range of diseases and applications [4]. “Diagnostic discovery” describes the process by which potential diagnoses identified by academic researchers but not flagged by the GE/GMC pipeline could be returned to GMCs, using an online researcher-identified potential diagnosis (RIPD) form. This would prompt the GMC to reanalyze the case on the updated pipeline with the researcher-identified variant, embedding researcher discovery into the diagnostic process (Fig. S1).

Given the substantial investment in sequencing and data storage required for clinical GS, assurance that the clinical pipeline can efficiently identify clinical grade molecular diagnoses is critical. This task is challenging in the context of diverse diseases, given the extensive and complex nature of human genome variation (encompassing single-nucleotide variants [SNVs], small indels, copy-number variants [CNVs], and structural variants [SVs]) [10, 11]. Here, we have used craniosynostosis (CRS), the premature fusion of one or more cranial sutures [12], as a model disorder to examine the performance of the 100kGP pipeline, by comparison with findings from intensive scrutiny of the data in the research environment aimed at generating a “truth data set”.

Several characteristics make CRS a suitable phenotype for this approach. First, CRS is relatively common (~1 in 2,000 live births) [13], constituting a primary rare disease recruitment category in 100kGP. Second, CRS is clinically and etiologically heterogeneous, with environmental [14], polygenic [15, 16], and monogenic/chromosomal factors all contributing. In the Oxford birth cohort of 666 individuals with CRS requiring surgery [17], 24% had an identifiable genetic cause, either monogenic (22%) or chromosomal (2%); 63% of patients with fusion of more than one cranial suture and/or associated syndromic features (including a positive family history) had an identified genetic cause, indicating that these clinical categories merit prioritization for genetic investigation. Third, 84% of the monogenic component could be screened out by testing just six [17] (now seven) [18, 19] commonly implicated genes; this testing was already widely available in the NHS, so that most facile molecular diagnoses had already been made prior to GS. Fourth, a previous study of CRS with suspected genetic cause but negative on routine genetic testing found that exome or genome sequencing yielded a substantial (37.5%) uplift in genetic diagnoses [20].

Importantly, CRS is characterized by a long “tail” of rare genetic diagnoses. In the Oxford survey [17], pathogenic variants in 20 rarely involved genes accounted for 23/666 (3.5%) of all cases, and in the exome/genome sequencing study [20], the 15 new diagnoses were identified in 14 different genes. A recent study from Norway reported similar findings [21]. As we expect the patients enrolled into 100kGP to be enriched for rare genetic causes, this heterogeneity presents a substantial challenge for pipeline-based diagnosis, so we considered that CRS could represent a stringent test of how well the GE/GMC pipeline worked. This work demonstrates the substantial benefit of exploiting specialist research expertise to augment the overall diagnostic rate in 100kGP, and indicates ways in which the diagnostic pipeline could be improved.

## MATERIALS AND METHODS

### Craniosynostosis disease cohort

The clinical protocol for 100kGP was approved by East of England–Cambridge South Research Ethics Committee (14/EE/1112). Written informed consent to obtain samples for genetics research was obtained. Patient recruitment for CRS required (1) the presence of multiple suture fusions and/or (2) additional clinical features or positive family history, indicating a syndrome; previous genetic testing for common causes of CRS and, if syndromic, normal chromosomal microarray, were also required (see Box S1 for details). Peripheral blood samples were obtained by venipuncture and DNA extracted for sequencing on Illumina instruments. Whenever possible, sporadically affected cases were sequenced as trios with their unaffected parents.

In 51 of the 114 families recruited, written informed consent had previously been obtained by researchers in the Clinical Genetics Group, Oxford (CGG) to investigate genetic causes of CRS (Oxfordshire Research Ethics Committee B [C02.143] and London–Riverside Research Ethics Committee [09/H0706/20]). This enabled independent molecular confirmation of some diagnoses by the CGG.

### Tiering pipeline

The pipeline used by GE/GMC to prioritize small variants (SNVs and indels <50 base pairs) into tiers is summarized in Box 1 [22]. Genomes were interrogated as family units; algorithms including frequency in control populations, mode of inheritance, appropriate segregation, effect on protein coding, and genotype–phenotype association were used to assign variants into four categories (tiers 1–3, with tier 1 the highest ranked, and “tier null” for the remainder), using complete or incomplete penetrance modes according to clinical indication [22]. This information was intersected with curated gene panels in PanelApp (applied depending on the clinical indication and phenotype data for each participant), prioritizing variants in diagnostic grade (“green”) genes (Box 1) [9]. Part-way through the program (Data Release V7, 25/07/19), Exomiser (comprising a suite of algorithms using random-walk analysis of protein interaction networks, clinical relevance, and cross-species phenotype comparisons) [23] was incorporated as an additional tool to rank potentially pathogenic variants based on frequency, predicted pathogenic impact, inheritance, and phenotype match. GMCs validated the prioritized results experimentally (usually by dideoxy-sequencing), and closed the case once assessment was complete. Importantly, GMCs were only mandated to examine all tier 1 and 2 variants, whereas examination of the longer list of tier 3 variants and Exomiser hits was discretionary, with variable effort (Box 1) [5]. Addition of new genes to the green category in PanelApp did not automatically trigger reassessment of closed cases.

CNV calls produced by Canvas software [24] were introduced into the pipeline in January 2019, but were not implemented on closed cases. The pipeline reported CNV calls >10 kb with a call quality score >10, and annotated and displayed CNV calls from the proband without considering mode of inheritance. Calls were assigned tier A if the CNV overlapped with a pathogenic region in a green gene in a panel applied to the patient (Box 1). In contrast to small variant tiering, a heterozygous CNV encompassing a biallelic gene would be tiered. Tier null CNVs were those that did not meet the criteria for tier A reporting.

Box 1 Genomics England tiering overview and assignment of all researcher-identified potential diagnosis (RIPD) alleles (*n* = 25) to tiers by PanelApp

**Table:** 

	Number of RIPD alleles in tier
**Tier 1**: Should be clinically assessed by Genomic Medicine Centres (GMCs). Includes high impact variants (e.g., likely loss of function) and de novo moderate impact variants (e.g., missense) within a curated list of green genes available through PanelApp with sufficient evidence associating them with the patient’s phenotype(s).	1^a^
**Tier 2**: Should be clinically assessed by GMCs. Includes moderate impact variants (e.g., missense) within a curated list of green genes available through PanelApp with sufficient evidence associating them with the patient’s phenotype(s).	1^a^
**Tier 3**: It is not expected that GMCs will review all of the variants in tier 3. For plausible candidate variants identified in genes *outside* of known disease gene panel(s), caution should be used during clinical assessment and interpretation. Includes high and moderate impact variants outside of the curated list of genes that are associated with the patient’s phenotype(s). Although most tier 3 variants will *not* be pathogenic, sometimes the causal variant will lie within tier 3. This could occur because there is insufficient evidence to support the inclusion of the gene within the relevant panel(s) at the time of analysis, or because the relevant panel was not applied.	12
**Tier A**: Copy-number variant (CNV) calls identified by Canvas, >10 kb size and with a call quality score >10, overlapping with a diagnostic grade gene in a panel applied to the patient.	1
**Tier null/untiered**: All variants not belonging to one of the categories above.	10^b^

^a^The biallelic variants in *MAN2B1* comprised one classified as tier 1 and one as tier 2.

^b^Both *MEGF8* alleles were untiered.

### Audit of GE/GMC-reported variants

Probands were identified by searching the Clinical Variant Ark (a restricted-access NHS database detailing all cases, variants, and phenotypes reported from 100kGP) for participants recruited with the clinical indications “CRS syndromes” or “CRS syndromes phenotypes." Phenotype data, applied gene panels, their iterations, and case status information were collected for each participant. Cases lacking CRS-related terms in the associated Human Phenotype Ontology (HPO) data [25] were excluded. For each case we determined whether the GMC had established a pathogenic or likely pathogenic variant, according to ACMG/AMP criteria [26], which we considered established a molecular diagnosis.

### Researcher-identified potential diagnoses (RIPDs)

The research-based analysis was performed by the CGG, through membership of the musculoskeletal GE Clinical Interpretation Partnership (Research Registry projects 65 and 365). Data were accessed within the GE Research Environment. The CGG considered reasons why variant(s) may not have been prioritized by the GE/GMC pipeline, and interrogated the data accordingly. The reasons identified were classified into four categories (1–4), as summarized in Box 2. To reduce the search space, variants were usually required to exhibit segregation concordant with the phenotype in the family (complete penetrance). The inheritance of each variant was separately annotated into one of five categories (A–E; Box 2), so that each RIPD could be classified with a number–letter combination. Detailed methods used to interrogate the data are provided in the Supplementary Information.

Following the detection of a putatively pathogenic variant by the CGG, a RIPD form was submitted to GE; in some instances, the case was still undergoing review by the GMC, whereas in others, it had already been closed with no primary findings. Genomics England then re-identified the patient and returned the variant to the recruiting GMC for review and reanalysis on the current, updated pipeline. The outcome of each GMC review of the RIPD was recorded in Clinical Variant Ark (Fig. S1). In four additional instances judged by the CGG to be of research interest but likely falling short of the threshold for clinical diagnosis, a “contact clinician” request was submitted instead of the RIPD; these cases are not discussed, as our focus here is on the diagnostic pipeline rather than novel findings.

Box 2 Classification of 18 alleles from 16 pathogenic/likely pathogenic researcher-identified potential diagnoses (RIPDs), according to reason missed by 100kGP pipeline and mode of inheritance

**Table:** 

**Category** ^**a**^	**Reason missed by 100,000 Genomes Project (100kGP)**	Number of RIPD alleles in category
1	Single-nucleotide variants (SNVs)/indels in PanelApp genes that had been missed or filtered out by the variant caller	6^b^
2	Variants in known developmental genes not rated green in the PanelApp for craniosynostosis (CRS) (± additional panels applied), at the time of the GMC’s analysis. To broaden the search space we scrutinized genes listed in G2P^DD 29^ and/or prioritized by Exomiser [23], and checked recently published medical literature for citations to additional candidate genes identified	7
3	Copy-number variants (CNVs) or structural variants (SVs) annotated using one or both of the callers applied to the GEL data, i.e., Canvas (CNV) and Manta (CNV/SV)	3
4	Genes for which apparently pathogenic variants of a particular class were present in two or more unrelated individuals, whereas variants with similar predicted pathogenic effect were rare in gnomAD (classified as *research genes*)	2
	**Mode of inheritance** ^a^	
A	Sporadic case associated with de novo mutation (DNM) in the proband	10^c^
B	Sporadic case with autosomal recessive (homozygous or compound heterozygous) inheritance	4
C	Ultrarare pathogenic variant in a singleton	1
D	Affected parent and child with concordant segregation of ultrarare genotype (dominant inheritance)	2^d^
E	Incorrect disease segregation model applied, owing to phenocopies or nonpenetrance	1

^a^Clinical Genetics Group, Oxford (CGG) researchers considered additional segregation (for example, affected sib pairs arising from biparental [autosomal recessive] inheritance or parental gonadal mosaicism for a DNM) and pathogenic molecular mechanisms (for example, cryptic splicing abnormalities), but if no convincing pathogenic example was found, no number category is assigned here.

^b^The missense allele in *MMP21* was detected, but only assigned to tier 3 because the other allele was filtered out.

^c^The *ERF* deletion was present in mosaic state in the unaffected father.

^d^The *HOXC* duplication was present in mosaic state in the affected father.

## RESULTS

### Patient composition and diagnostic summary

**Fig. 1:**
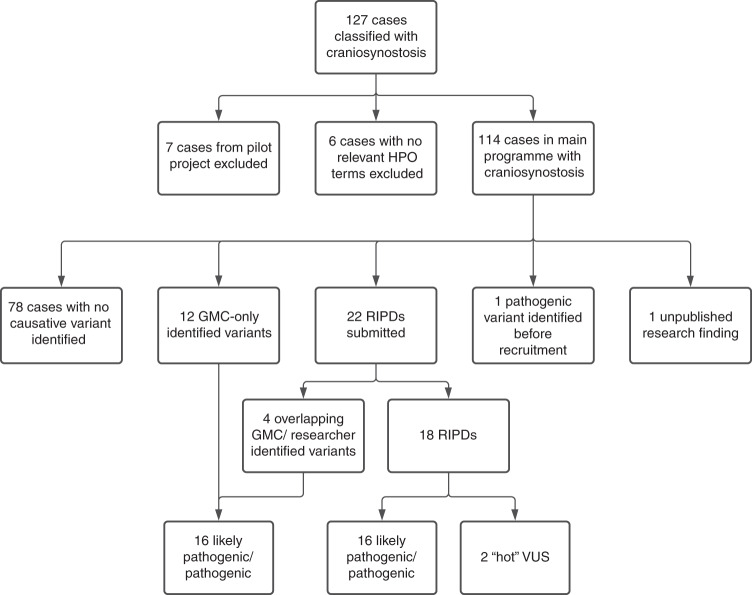
Summary of craniosynostosis (CRS) cases and outcomes. One hundred twenty-seven cases with CRS were identified from the Clinical Variant Ark search, reduced to 114 after exclusion of participants recruited to the 100kGP Pilot project, and participants with no definite CRS-related phenotype terms. Potentially diagnostic variants have been identified in 36 cases thus far. Seventy-eight remaining cases have either been closed with no primary findings (*n* = 75) or are awaiting Genomic Medicine Centre (GMC) review (*n* = 3).

In total, 127 families primarily classified with CRS were recruited to 100kGP (Fig. 1). We excluded seven families from the Pilot phase [27], as their data were not available in the Research Environment; in an additional six families, no CRS phenotypes were annotated in the associated HPO terms. Hence, we focused on 114 bona fide CRS families in the main program, including 15 families with more than one affected individual, and 72 sporadically affected probands analyzed as parent–child trios (Table S1). Eighty-two of the probands (72%) were classified as having a syndromic clinical presentation and 53 (46%) had fusion of multiple cranial sutures (Table S2). To date, GMCs have autonomously confirmed molecular diagnoses in 16 cases (14.0%), RIPDs have independently provided diagnoses in 16 cases, and two diagnoses came from other sources (one pathogenic variant identified before 100kGP recruitment, and one unpublished research finding [Fig. 1, Table 1, Table S3, Table S4]), yielding an overall diagnosis rate of 34/114 (29.8%).

**Table 1:** Researcher-identified potential diagnoses (RIPDs) submitted by Clinical Genetics Group, Oxford (CGG) for patients with craniosynostosis (CRS) recruited to the 100,000 Genomes Project (100kGP)^a^.

Case	Researcher category (Box 2)	Panels applied in addition to CRS	Gene	cDNA change	Protein change	Tier	Exomiser rank	Inheritance	Gene green on original/updated panel?	Pathogenicity	Also identified by GE/GMC?	Currently identifiable by NHSE pipeline?
**Tier 1, 2 or A variants**												
1	N/A	5	*MAN2B1*	c.[1830+1G>C];[2248C>T]	p.([?]); ([Arg750Trp])	Tier 1; tier 2	2	Recessive	Original	Pathogenic	Y	Y
2	N/A	10	3.4 Mb Chr 6 del	−	−	Tier A	Unranked	De novo	Original	Pathogenic	Y	Y
**Monoallelic tier 3 variants**												
3	N/A	0	*KMT5B*	c.557T>A	p.(Leu186*)	Tier 3	1	De novo	No	Pathogenic	Y	Y
4	2A	1	*SMAD2*	c.1223T>C	p.(Leu408Pro)	Tier 3	2	De novo	No	VUS	N/A	N/A
5	2A	0	*SMAD6*	c.40T>C	p.(Trp14Arg)	Tier 3	1	De novo	Updated	Likely pathogenic	N	Y
6	2A	0	*CDK13*	c.2563G>C	p.(Asp855His)	Tier 3	2	De novo	No	Likely pathogenic	N	Y
7	2A	7	*HNRNPK*	c.1291G>T	p.(Glu431*)	Tier 3	1	De novo	Updated	Pathogenic	N	Y
8	2A	1	*FBXO11*	c.2731_2732insGACA	p.(Thr911Argfs*5)	Tier 3	3	De novo	Updated	Likely pathogenic	N	Y
9	4A	1	*SOX6*	c.242C>G	p.(Ser81*)	Tier 3	2	De novo	No	Pathogenic	N	Y
10	4C	1	*SOX6*	c.277C>T	p.(Arg93*)	Tier 3	63	Parents not available	No	Likely Pathogenic	N	N
11	2A	0	*BRWD3*	c.4012C>T	p.(Gln1338*)	Tier 3	1	De novo	No	Pathogenic	N	Y
12	2A	1	*PTCH1*	c.290del	p.(Asn97Thrfs*20)	Tier 3	1	De novo	No	Pathogenic	N	Y
13	2A	1	*ALX1*	c.541C>A	p.(Gln181Lys)	Tier 3	5	De novo	No	VUS	N/A	N/A
**Untiered small variants**												
14	1B; 1B	1	*MEGF8*	c.[4496G>A];[7766_7768del]	p.([Arg1499His]); ([Phe2589del])	Both untiered	96; unranked	Compound heterozygous	Original	Likely pathogenic/likely pathogenic	N	N
15	1B; 1B	3	*MMP21*	c.[671_684del];[775C>G]	p.([Val224Glyfs*29]); ([His259Asp])	Untiered; tier 3	Both unranked	Compound heterozygous	Original	Pathogenic/likely pathogenic	N	Y
16	1A	1	*ARID1B*	c.3594delinsCCCCCA	p.(Gly1199Profs*14)	Untiered	Unranked	De novo	Original	Pathogenic	N	Y
17	2A	1	*TRAF7*	c.1885A>G	p.(Ser629Gly)	Untiered	3	De novo	Updated	Likely pathogenic	N	Y
18	1E	1	*TCF12*	c.1870C>T	p.(Leu624Phe)	Untiered	Unranked	De novo	Original	Pathogenic	N	Y
19	N/A	3	*OGT*	c.539A>G	p.(Tyr180Cys)	Untiered	1	De novo	Updated	Pathogenic	Y	Y
**Untiered copy-number and structural variants**												
20	3D	0	13.4 Mb Chr 7 inv (*TWIST1*)	−	−	Untiered	Unranked	Dominant (proband, affected mother)	Original	Pathogenic	N	N
21	3A	1	314 kb Chr 19 del (*ERF*)	−	−	Untiered	Unranked	De novo (mosaic in unaffected father)	Original	Pathogenic	N	Y
22	3D	2	285 kb Chr 12 dup	−	−	Untiered	Unranked	Dominant (mosaic in affected father)	No	Likely pathogenic	N	N

*cDNA* complementary DNA, *GE/GMC* Genomics England/Genomic Medicine Centre, *N/A* not applicable, *NHSE* NHS England, *VUS* variant of uncertain significance.

^a^For a more detailed version of the content of this table, please see Table S4.

### GMC-identified variants

Sixteen variants (in cases 1–3 and 19 in Table 1 and 23–34 in Table S3) were classified by GMCs as likely pathogenic or pathogenic. In 13/16 cases, the causative variants were identified from tier 1/2 or tier A data (Box 1). Of the remaining three variants, the *KMT5B* de novo variant (case 3) was found in tier 3 data, while the X-linked *OGT* variant in case 19 and the de novo *ZBTB20* variant in case 34 were untiered but were identified because the respective GMC had searched the Exomiser [23] data.

### RIPDs

Twenty-two RIPDs were submitted by the CGG (Fig. 1), of which 20 (comprising 22 variants; 18 monoallelic and 2 biallelic) were either tier 3 or untiered. The outcome of assessment and validation of each RIPD by the GMC is summarized in Table 1. In four cases (1–3 and 19), the variant was independently reported as pathogenic by the GMC; these are not discussed further. From the remaining 18 “researcher-only” RIPDs, 16 cases (comprising 18 variants) were classified as pathogenic/likely pathogenic and two were reported as VUS.

### Monoallelic tier 3 variants

Ten of 18 researcher-only RIPDs (cases 4–13) were monoallelic tier 3 variants that were not tier 1/2 because the gene was not diagnostic grade (green) on the panel(s) applied at the time of analysis. While for three cases (5, 7, 8) the genes are now diagnostic grade on at least one relevant panel, no process currently exists for GMCs routinely to reanalyze cases on updated panels. The remaining seven tier 3 RIPDs are variants in genes that are still not rated diagnostic on the panels applied to the patient. However, most are still likely to be contributing fully or partially to the patient’s phenotype. All genes except *SOX6* (which we distinguish as a “research gene” because the two cases [9, 10] contributed to the original discovery cohort) [28] were already known to harbor pathogenic variants contributing to developmental disorders [19, 29]. Notably 9/10 monoallelic tier 3 variants (excepting case 10, for whom parental GS was not available) arose as de novo mutations (DNMs) in sporadically affected cases analyzed as parent–child trios; these nine were all ranked within the top five candidates by Exomiser. Combining all available evidence, two variants were classified as VUS, four as likely pathogenic and four as pathogenic (Table 1, Table S4).

### Untiered small variants

Five researcher-only RIPDs (cases 14–18) were submitted for cases including an untiered SNV or indel (Table 1, Table S4). Cases 14 and 15 both harbored biallelic variants in diagnostic grade genes (*MEGF8*, *MMP21*) on one of the panels applied, but in each case one of the variants was a heterozygous deletion (of 3 or 14 nucleotides, respectively) that had been filtered out based on quality settings. In each case the second variant, a heterozygous missense, was not specifically flagged, even though the patient had a very characteristic phenotype (*MEGF8*: Carpenter syndrome; *MMP21:* heterotaxy) associated with a limited number of known disease-causing genes. Case 16 harbors a de novo indel in *ARID1B* (deletion of 1 nucleotide and insertion of 6 nucleotides) that was also filtered out during variant quality control. In case 17, a de novo variant in *TRAF7* (ranked 3 by Exomiser) was filtered out from tiering because 1 of 32 reads in the mother appeared to match the child’s variant; inspection in the Integrative Genomics Viewer (IGV) [30] suggested this was caused by a low quality read, as a nucleotide two residues away was also miscalled. The family in case 18 comprises three affected male siblings with differing cranial phenotypes; in one sibling with bicoronal synostosis, a de novo variant in *TCF12* was reported as an RIPD. This variant had in fact been identified prior to submission to 100kGP in a panel screen, and had been classified as pathogenic; however, within 100kGP, it had been missed both in tiering and by Exomiser, because the analyses assumed that the three siblings must share the same genetic pathology.

### Copy-number and structural variants

Three researcher-only RIPDs (cases 20–22) were untiered SV/CNV, comprising a complex inversion involving *TWIST1* (case 20), deletion including *ERF* (case 21) [31] and duplication involving the *HOXC* gene cluster (case 22), each of which was detected by the CGG using overlapping Canvas [24] and Manta [32] calls (Table 1, Table S4, and Supplementary Information). While analysis of CNVs using the Canvas caller is now incorporated into the GE/GMC pipeline, cases analyzed before January 2019 did not have tiered CNVs. As *TWIST1* and *ERF* are diagnostic grade genes for CRS, the rearrangements were retrospectively analyzed on the updated GE pipeline. Although the *ERF* deletion was called as tier A, the *TWIST1* inversion was still missed because the breakpoints flanked the gene. The *HOXC* duplication was associated with a distinctive craniofacial phenotype resembling a published mouse mutant [33] and classified as a research finding.

### Additional diagnoses

Two diagnoses that were neither found by the GE/GMC pipeline nor submitted as RIPDs are summarized in Table S3. An individual (case 35) with the clinical features of Simpson–Golabi–Behmel (SGB) syndrome previously had targeted testing of *GPC3*, and a deletion of exons 7 and 8 was reported. The patient was referred to 100kGP by a clinician unaware of the rare association of SGB syndrome with CRS; this case was analyzed with CNVs on the 100kGP pipeline, however as *GPC3* was not a diagnostic grade gene in the panels applied, the CNV was not called and a negative report issued. In case 36, an affected mother and child, members of a four-generation family affected by CRS, had GS by 100kGP. Independent investigation by the CGG had previously revealed a segregating 11.5-kb duplication in a noncoding region of chromosome 1p31.3, which was not tiered by GE. This was shown to be causative based on mouse modeling (unpublished).

## DISCUSSION

Using CRS patients recruited to the 100kGP as an example, we sought to measure the added value from scrutiny of GS data by a research team, compared to the clinical pipeline. From 22 submitted RIPDs, 16 additional researcher-only diagnoses were confirmed by GMCs as likely pathogenic or pathogenic, doubling the number of diagnoses from 16 to 32. An additional two diagnoses were made outside the GMC/RIPD reporting systems; hence the diagnostic sensitivity of the GE/GMC pipeline for CRS was only 47% (16/34), considerably lower than the overall 77% figure suggested by the 100kGP pilot [27]. The final rate of diagnoses for CRS from the 100kGP was 29.8% (34/114), with a much higher success rate for syndromic (39.0%) than nonsyndromic (6.25%) presentations (Table S2; Fisher’s exact test one-tailed *P* = 0.0003). In the context of CRS, this work demonstrates the substantial uplift that expert researcher-led examination of GS data can contribute to clinical grade molecular diagnoses.

**Fig. 2:**
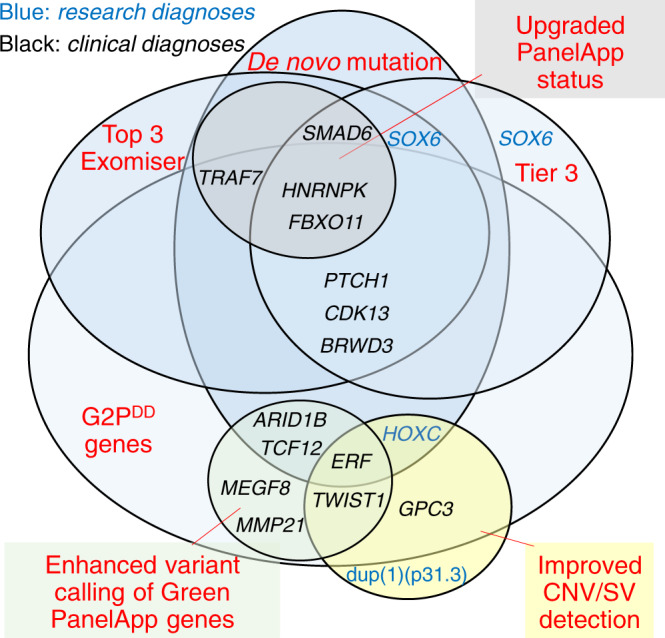
Improved approaches to identifying diagnostic variants in craniosynostosis. Venn diagram classifying each of 16 researcher-identified potential diagnoses (RIPDs) considered diagnostic (excluding variants of uncertain significance [VUS], and those independently found by Genomic Medicine Centres [GMCs]) and 2 additional cases, according to methods that would have identified them.

A major goal of this study was to use the insights from researcher-identified diagnoses to highlight ways to improve the clinical pipelines. We summarize in Fig. 2 the major features of the missed diagnoses, to signpost which approaches would have detected them.

In evaluating how this information could be implemented in diagnostic GS, we recognize that the search effort in a clinical setting needs to be substantially less intensive than might be feasible in a research laboratory. This requires balancing the conflicting demands of high sensitivity (recall), which minimizes false negative calls, and high precision (positive predictive value), which minimizes false positive calls. It is evident that exclusive use of a panel-based approach (PanelApp) with the aim of maximizing precision was inadequate, because, even with optimal application (incorporating recent updates to PanelApp, adding 4 diagnoses; and optimizing variant calling, adding 6 diagnoses; see Fig. 2), the sensitivity achieved would still only be 76% (26/34), with 4 additional clinical diagnoses (variants in *BRWD3*, *CDK13*, *GPC3*, and *PTCH1*) continuing to be missed. A comprehensive approach would be to consider as candidates *all* validated genes mutated in developmental disorders (for example, confirmed/green genes from G2P^DD^ [DDG2P] lists) [29]; while this would overall add 14 diagnoses (sensitivity 88%), the workflow would be very laborious owing to the large number of genes to scrutinize (currently 2,149 in G2P^DD^), which would generate many false positive calls hence reducing precision.

An approach that balances the joint requirements of high sensitivity and precision is suggested by the observation (Fig. 2) that 10 of the additional clinical diagnoses are single-nucleotide or indel-associated de novo variants; systematic scrutiny of DNMs would have increased sensitivity by 29% to 76% (26/34) with modest additional analysis burden, because fewer than two protein-altering DNMs are expected per genome [34]. This approach (combining panels with DNMs) harmonizes with draft NHS England reporting guidance for enhanced analysis of GS data [35]; scrutiny of the top 3 Exomiser hits, which is also mandated by this guidance, would yield substantially overlapping information (Fig. 2). In the 100kGP Pilot, Exomiser-based prioritization was shown to yield a 19% enhancement over panels [27].

We identified two further key factors eroding the overall diagnostic sensitivity for CRS in the 100kGP program: incorrect filtering out of SNVs/indels (5 cases), and difficulties with prioritizing causative SV/CNVs (5 cases). In combination, this led to a loss of 10/34 (29%) of all diagnoses (Fig. 2). We observed three instances (cases 14, 15, 16, Table 1, Table S4) in which multinucleotide indel calls were mistakenly filtered out. Other dropouts were caused by poor quality parental variant reads (case 17), and forcing a specific segregation model on a multiply affected sibship (case 18). Four probands (cases 20, 22, 35, and 36) had pathogenic CNVs/SVs that would be missed, even by the updated GE/GMC pipeline that intersects Canvas-based calling with green PanelApp genes (however we classified two as research rather than clinical diagnoses). Of note, the Manta output, which both complements and augments Canvas data, was not utilized for clinical CNV/SV calling. Given the structural complexity of the human genome and the inbuilt limitations of short-read sequencing technology (which yields CNV/SV calls of poor specificity and unpredictable sensitivity) [36], optimized clinical CNV/SV calling represents a key target for methodological improvements, essential for leveraging the full added value from sequencing genomes compared to exomes.

While the use of HPO terms for clinical classification has major benefits, reliance to the exclusion of clinical acumen has drawbacks. Case 14 had a clinical diagnosis of Carpenter syndrome, an autosomal recessive disorder with a very restricted spectrum of disease-associated genes. However, this diagnosis was not recorded in 100kGP data and neither of the two contributing variants in *MEGF8* was tiered. Flagging of previously reported pathogenic alleles in recessive disorders relevant to the phenotype [37] would have triggered intensive search for a second damaging variant. Along similar lines, the *GPC3* deletion (case 35) was missed because PanelApp interrogation was based on HPO terms, rather than on the information that the clinical diagnosis was SGB syndrome.

Our findings show that to optimize molecular diagnosis from GS data, the active engagement of research laboratories is essential. Unfortunately this cannot be relied upon, owing to multiple factors including (1) the patchiness of research efforts across different clinical disorders, (2) potential lack of perceived priority in research laboratories to identify and/or communicate clinical diagnoses, and (3) reluctance of research-funding bodies to invest monies into what appears to be diagnostic, rather than research activity. For GS-based diagnostics in the UK, this work has important implications for the new NHS Genomic Medicine Service [38], in which subjects can choose to opt in or out of additional research being performed on their data. The precise means by which the “research question” is presented to the patient/family, in terms of the written information and consenting process, will have material effect on the proportion of patients/families in which further diagnostic discovery would be feasible from their GS data.

The large number of researcher-only diagnoses that involve variants in genes (*n* = 10) not green-listed on the CRS panel is not surprising [17, 21]. This wide genetic spectrum likely reflects the pathogenesis of cranial suture fusion, whereby some genes that are recurrently mutated directly perturb intrinsic suture function [39], whereas for more rarely mutated genes, the mechanism may be more nonspecific, for example by predisposition to macrocephaly (which may trigger CRS in a restricted intrauterine environment), or by perturbation of the poorly understood interactions between brain enlargement and growth at the cranial sutures [39, 40]. Four of the genes identified (*GPC3*, *PTCH1*, *SOX6*, *TRAF7*) are now amber or red-listed in PanelApp, and pathogenic variants in *ARID1B*, *CDK13*, *FBXO11* and *HNRNPK* have also been associated with CRS in a small number of cases (Table S5). We are not aware of previous descriptions of CRS associated with variants in *BRWD3* or *MMP21*, but the other clinical features in these cases, in combination with the associated variants identified, were considered sufficient to assign pathogenic or likely pathogenic status. CRS may represent an extension of previously described phenotypes, the frequency of which will become evident as each pathological entity is better delineated.

Identification of several of the variants has led to new molecular diagnostic insights, as illustrated by publications on *SMAD6* [19], *SOX6 *[28], and *ERF* [31]; additionally, the partial duplication of the *HOXC* cluster (case 22) gives rise to an apparently novel combination of phenotypes. Many other discoveries from the combined clinical research approach have been reported in other disease domains of 100kGP [27].

Our analysis of CRS may not be representative of 100kGP data overall. Craniosynostosis likely represents a stringent test of the GS pipeline, given the extensive prior molecular and phenotypic screening undertaken before case recruitment (Box S1), and because CRS is known to be associated with a long tail of rare genetic diagnoses [17, 21]. The reliance of GE/GMC on a panel-based diagnostic approach was evidently not well suited to this scenario. Nevertheless this “truth” data set provides test cases to evaluate future improvements to the NHS pipelines, as well as valuable insights into ways to optimize implementation of clinical GS more generally.

## Supplementary information

Supplementary Materials

Supplementary MaterialsTable S3

Supplementary MaterialsTable S4

## Data Availability

Primary data from 100kGP, which are held in a secure Research Environment, are available to registered users. Please see https://www.genomicsengland.co.uk/about-gecip/for-gecip-members/data-and-data-access/ for further information.
