# Supplementary material for: Evaluating the performance of a clinical genome sequencing programme for diagnosis of rare genetic disease, seen through the lens of craniosynostosis
Source: Genet Med. Author manuscript; Available in PMC 2021 Dec 2. (PMC8629760; doi:10.1038/s41436-021-01297-5)
Supplement: Supplementary Information [file EMS138012-supplement-Supplementary_Information.docx]

**Supplementary Information**

**Contents**

# **Supplementary Methods: Variant detection by the Oxford Clinical Genetics Group**

Small variants (single nucleotide variants [SNVs] and indels) 2

Structural and copy number variants (SV/CNV) 3

**References** 9

**Box S1**. Eligibility criteria required for inclusion of patients with CRS in 100kGP 10

**Fig. S1**. Relationship between GE/GMC and Researcher pipelines for clinical diagnosis 11

**Table S1**. Composition of the 100kG CRS cohort 12

**Table S2**. Patterns of suture fusions in the 100kG CRS cohort 13

**Table S3**. GE/GMC diagnoses (cases 23-34) and additional diagnoses (cases 35, 36)

in patients recruited to the 100kGP CRS cohort [available as separate Excel table]

**Table S4**. Researcher-identified potential diagnoses (RIPDs) submitted by CGG for

patients with CRS recruited to 100kGP [available as separate Excel table]

**Table S5**. Case reports describing CRS associated with mutations in genes identified

in the RIPD cohort 14

# **Supplementary Methods: Variant detection by the Oxford Clinical Genetics Group**

**Small variants (single nucleotide variants [SNVs] and indels)**

***De novo* mutations (DNMs):** All analyses were performed in the Genomics England (GE) Research Environment. Enhanced DNM analysis was the single most powerful way to increase diagnostic yield.

First, DNMs not ranked Tier 1 or Tier 2 (and therefore Tier 3) were found in tiering data by filtering on DNM annotation. These calls were intersected with genes listed in G2P^1^ and/or reported in recent literature, to generate a shortlist of DNM calls for individual scrutiny.

Second, we analysed the top 20 Exomiser hits^2^ for all 114 individuals. Exomiser candidates are scored on rarity, pathogenicity (generated by Polyphen2, SIFT and MutationTaster) and similarity of the patient phenotype (reported by assigned HPO terms) to phenotypes listed in OMIM and Orphanet rare diseases known to be associated with the gene or neighbouring genes in the StringDB database, and mouse and zebrafish models. We found that DNMs of potential pathogenic significance scored in the top 5 ranking (Table S4).

Third, multi-sample variant call format (VCF) files for families enrolled as trios were passed through the Platypus ‘bayesianDeNovoFilter.py’ script under default parameters.^3^ The modified multi-sample VCF was annotated with base and stringent filters (see <https://research-help.genomicsengland.co.uk/display/GERE/De+novo+variant+research+dataset>) and those annotated ‘PASS’ were extracted. All pass-variants were annotated with Variant Effect Predictor (VEP)^4^ and intersected with G2P data.^1^ Non-coding, intronic, synonymous, and benign variants were excluded from the dataset, leaving the remaining variants to be investigated manually. This led to the identification of the *TCF12* variant (case 18), which was missed by all the other methods.

Fourth, to identify DNMs that might represent novel associations with craniosynostosis (CRS), we intersected DNM calls with rare damaging variants identified in tiering data and VCF files. This revealed heterozygous nonsense variants of *SOX6* in two different families (one DNM, one sporadic case). These variants contributed to a publication delineating a novel *SOX6*-associated developmental syndrome;^5^ hence *SOX6* is classified as a research gene in the analysis.

**Indels:** A further category systematically missed by the GE/GMC pipelines were indels (cases 14-16 with an additional example (*TLK2*) found in a patient with CRS classified within the Intellectual Disability cohort of 100kGP). As such, insertion, deletion, and frameshift variants were called from participant VCF files, VEP annotated, and intersected with genes listed in the G2P dataset. Non-coding, intronic, and benign variants were removed, and a maximum allele frequency threshold was assigned at 0.000045 (based on previously reported frameworks)^6,7^ for autosomal dominant mode of inheritance (MOI), and 0.05 for recessive MOI. Indels identified in more than 5 unrelated families were removed, aiming to increase identification of rare, and more likely pathogenic, variants.

**Recessive/ X-linked Analyses:** All variants annotated as homozygous or hemizygous in the participant VCF were called and annotated with VEP. An allele-frequency based filter was used, considering an overall gnomAD frequency of less than 0.05. Remaining variants were investigated manually, considering factors such as likely pathogenicity (reported by Polyphen, SIFT, and CADD scores), functional consequence, conservation, and similarity of patient phenotype to reported literature. This analysis revealed a homozygous nonsense variant in *TMCO1* in a patient annotated under the normalized disease grouping of ‘Craniosynostosis syndromes’. This gene was missed by GEL/GMC as *TMCO1* was not Green in PanelApp at the time. The variant was reported by the CGG as a RIPD and later classified by the GMC as pathogenic. However, this variant was excluded from the data in the main paper because the proband lacked HPO terms for CRS.

**Structural and copy number variants (SV/CNV)**

All 100kGP data were called by GE using two SV/CNV callers, Canvas and Manta. We analysed the CRS cohort data files and later augmented these calls with Lumpy, an SV caller with orthogonal properties to Manta.


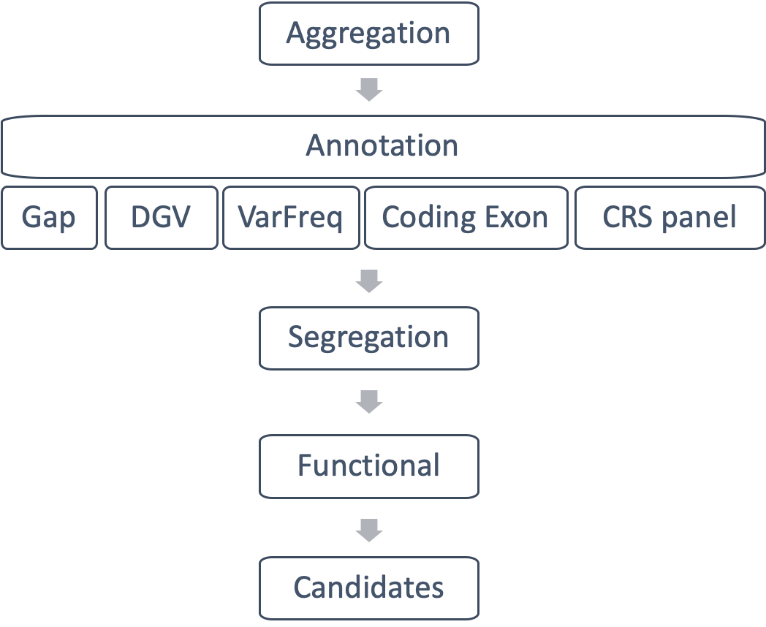


An analysis pipeline (**Supplementary** **Figure 1**) was designed to extract true and most likely pathogenic variants. A series of annotations was applied to the SV calls in order to (1) filter out likely common or false positive SV/CNV, (2) categorise SVs based on segregation analysis and (3) prioritise most likely true positive and pathogenic calls for further functional studies. As described below, five annotations (VarCount, DGV, gap region, Coding region, and a CRS gene panel) were applied to the Canvas and Manta datasets, and a Union annotation was added for the Lumpy dataset.

**Supplementary Figure 1.** Pipeline for identification of SV/CNV candidates


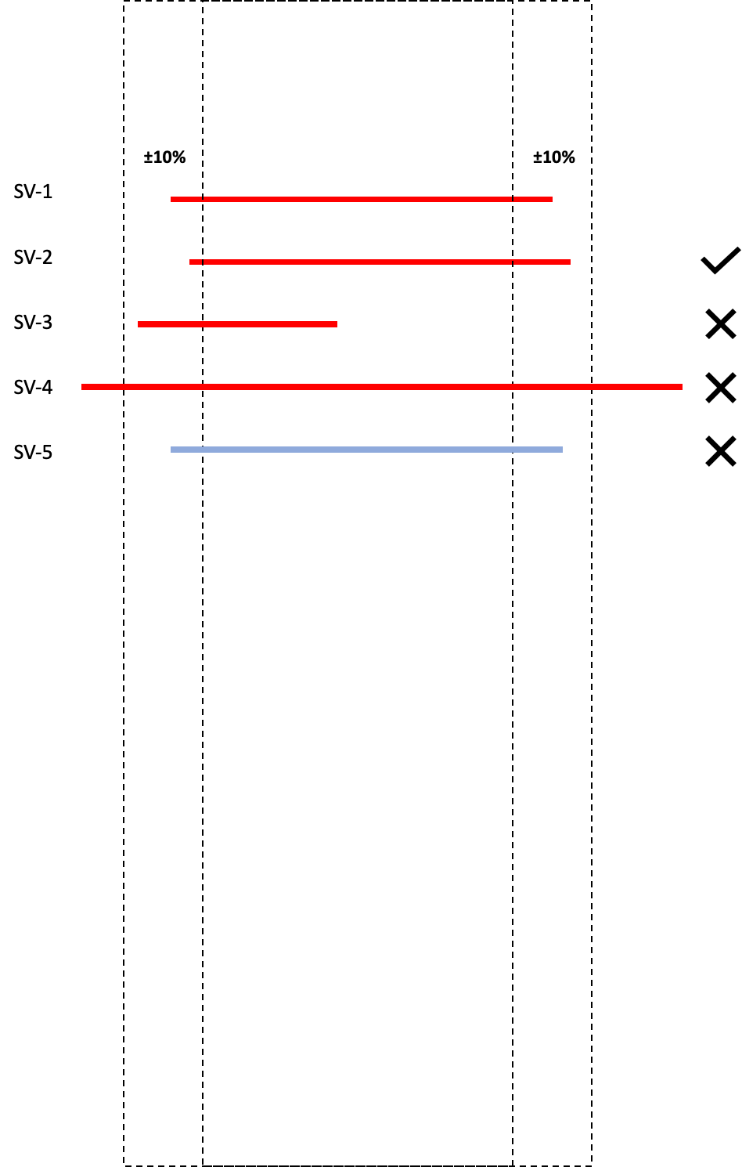
**VarCount:** This annotation aimed to filter out likely common or false positive SV/CNV based on the rarity of a specific call. The rationale is that high frequency variants are likely to be noise or common polymorphisms, while rare variants are of primary interest in our data. A particular challenge for this approach occurs when the same rearrangement is called with slightly differently breakpoints in different samples. Such calls are particularly likely to be associated with repetitive regions, assembly gaps, inversions, highly polymorphic regions, and most Canvas calls, since Canvas relies solely on a change in read depth to call CNVs. Therefore, we applied a “fuzzy-end” matching strategy which determines SV calls as the same event using five pieces of information: chr, start, end, SV size (SV length in base pairs), and SV type, as illustrated in **Supplementary Figure 2**.

**Supplementary Figure 2**. Soft "fuzzy" matching approach for identifying calls representing the same event in different samples. SV calls were determined as the same event if they share the same chr, SV type, and the start and end lie within a range that is 10% of the SV size in base pairs, such as SV-1 and SV-2. SVs (such as SV-3, SV-4, and SV-5 [a different event type]) that did not match all criteria were marked as representing different events.

Permissible length variation of ±10% was selected after testing 5%, 10%, 15%, and 20% of the SV size in the query. The results showed that 5% SV size was too short to match large events especially Mb-sized inversions (INV), whilst ≥15% SV size failed to distinguish small (~100 bp) yet unique SVs in the same region.

Using this strategy, each SV call was queried against the entire CRS cohort and the number of matching calls for each query was deposited as the VarCount value of the queried call.

**Database of Genomic Variants (DGV):** The DGV database aggregates SV/CNV in >22,000 genomes of the healthy population,^8^ and therefore, provides a valuable annotation for excluding common/benign SVs. An SV observed in DGV, hence in the healthy population, is highly unlikely to be pathogenic. Therefore, SVs were excluded if they were also observed in DGV. However, SVs were recovered regardless of DGV annotation when they intersected with any of the ClinGen Dosage Sensitivity - Recurrent CNVs.^9^ This ensured that no recurrent pathogenic CNVs escaped analysis owing to the stringent DGV filter.

DGV data were obtained from UCSC DGV Struc Var – dgvMerged. As for VarCount, a fuzzy query (±20% SV size) was applied to determine if an SV call was present in DGV, with a few adjustments to accommodate the unique features of DGV data. A large proportion of DGV SVs were obtained from chromosomal microarray data; these SVs are likely to have less well-defined breakpoints compared to the SV calls from genome sequencing (GS). The precision of the DGV matching is more relaxed than VarCount matching, because different SV/CNV events within a given genomic region are likely to be benign when this region harbours similar benign rearrangements in a healthy control (DGV) population. The ±20% SV size criterion was selected for the DGV query value after testing 10%, 15%, 20%, 25%, and 30%. In addition, to address the different annotation of variant annotation terminology in DGV compared with Canvas/Manta, we applied the conversions shown in **Supplementary Table 1**.

**Supplementary Table 1**. DGV to Manta/Canvas call conversions

|  | Manta/Canvas call type | | | |
| --- | --- | --- | --- | --- |
| DGV SV type | DEL | DUP | INS | INV |
| Complex | 🗸 | 🗸 | 🗸 | 🗸 |
| Gain + loss | 🗸 | 🗸 |  |  |
| Deletion/loss | 🗸 |  |  |  |
| Gain/duplication/tandem duplication |  | 🗸 |  |  |
| Inversion |  |  |  | 🗸 |
| Insertion/mobile element insertion/novel sequence insertion |  |  | 🗸 |  |

**Gap regions**: The Gap annotation aims to exclude noisy clusters at specific regions of the genome that are largely inaccessible by GS. These include the short arms from acrocentric chromosomes, heterochomatin, telomeres, centromeres, gaps between contigs and scaffolds in chromosome assemblies. These regions were obtained from the UCSC genome table browser under the gap subset.^10^ The SV calls were then queried against the gap subset using the midpoint of the call, as this gap annotation aims to remove FP calls clustered around noisy region.

**Coding regions**: This annotation allows SVs to be prioritised under the assumption that in general, protein altering SVs are more likely to be pathogenic compared to non-coding SVs. The coding region data were extracted from UCSC table browser gene & gene predictions (GENCODE v32),^11^ and the SV calls were annotated with the coding regions using an “intersection” query approach. In this approach, the SV break points were used when identifying an intersection between the SV and a coding region. As shown in **Supplementary Figure 3**, there are six possible relations between the SV (green) and a region of interest (blue). Of all possible relations, four are annotated as positive for SV-region intersection. SVs were prioritised when they intersected with at least one coding region.

**Supplementary Figure 3**. SV-coding region intersection criteria^a^


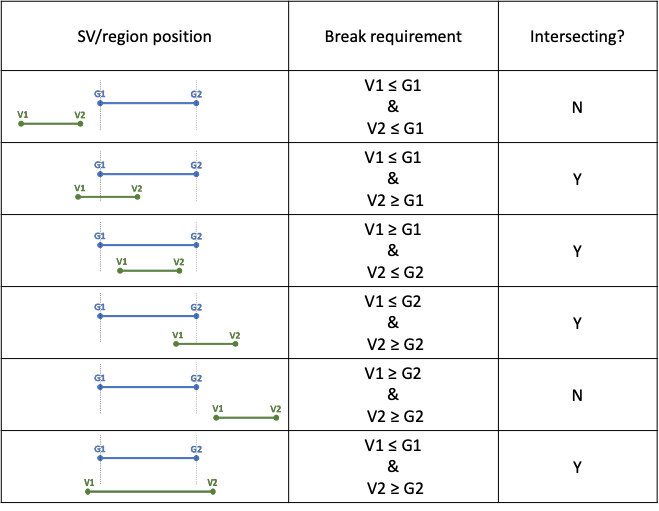


^a^V1/V2 represents the start/end of an SV; G1/G2 represents the

start/end of a region of interest (gene/exon)

**CRS panel**: SV calls were further annotated using a panel of known CRS genes. These genes were extracted from the Genomics England PanelApp Craniosynostosis (Version 2.2),^12^ and the respective genomic locations were defined by Ensembl Genes 101 GRCh38.p13/GRCh37.p13. Genes are mostly defined from the 5′-UTR to the 3′-UTR by Ensembl, while sometimes extensive regulatory regions are included (for example, *TWIST1* is defined by Ensembl to include a ~96 kb regulatory region upstream of the 5′-UTR). The CRS panel was queried against the SV calls using the intersection approach outlined in **Supplementary Figure 3**. SVs intersecting with any known CRS gene were prioritized.

**Lumpy concordant matches**: The Lumpy callset was annotated to identify concordant calls with Manta/Canvas. The Lumpy calls were queried against Manta/Canvas calls using the same “Fuzzy” matching approach as described in **Supplementary Figure 2**. From this annotation, the discordant calls aim to provide extra SVs due to the low sensitivity of Manta/Canvas, while the concordant calls provided more confident prioritisation for singleton samples without available segregation analysis.

**Segregation analysis:** Two overall types of segregation analyses were carried out under three different hypotheses:

(1) CRS phenotypes are caused by SVs that segregate with the phenotypes. Under this assumption, segregating SVs were selected, such as inherited SVs in familial cases and including *de novo* SVs in sporadic cases with unaffected parents.

(2) Pathogenic SVs may cause CRS through complex mechanisms, such as parental mosaicism or incomplete penetrance. For this hypothesis, non-segregating and yet compelling SVs were further analysed for evidence of pathogenicity through complex mechanisms. An example would be SVs affecting known CRS genes but inherited from an apparently unaffected parent.

(3) Pathogenic SVs may have been missed by the default callers. To test this hypothesis, discordant calls from Lumpy were analysed, with the rationale being that Lumpy may be able to detect some of the pathogenic SVs missed by Manta/Canvas.

**SV filtering and prioritisation**: SVs were filtered and prioritised using the annotations and segregation analysis described above, as summarised in **Supplementary Table 2**. Filtering and prioritization criteria were adjusted based on family structure and segregation pattern. The four filtering annotations provided hard exclusion criteria, whilst the three prioritisation strategies provided soft filters that reserved low priority SVs for further analyses when needed. For the caller concordance, all Manta&Canvas calls were analysed; concordant Lumpy calls were analysed for singletons while discordant Lumpy calls were analysed for all other cases.

**Further analysis**: These processes generated large lists of SVs, which required further manual examination to assess the evidence that they were real and to further assess pathogenicity.

1. To verify the SV calls, the Integrative Genomics Viewer (IGV)^13^ was used to assess supporting reads at the breakpoints; Samplot^14^ was used to visualize the overall read depth change supporting the called SV.

**Supplementary Table 2**. SV segregation, filtering, and prioritization strategies

| Segregation | SV Inheritance | Segregating | | | | Non-segregating | |
| --- | --- | --- | --- | --- | --- | --- | --- |
|  | Family structure^a^ | Sporadic Trio | Familial Duos | Complex | Singletons | Sporadic Trios | Sporadic Duos |
| Filtering | VarCount  (non-ClinGen)^b^ | = 1 | = 2 | = # of affected | = 1 | = 2 | |
|  | DGV | Not observed in DGV or intersecting ClinGen recurrent CNVs | | | | | |
|  | Gap | N | | | | | |
|  | Concordance^c^ | M&C + discordant L | M&C + discordant L | M&C + discordant L | M&C + concordant L | M&C + discordant L | M&C + discordant L |
| Prioritization | Coding regions | Coding SVs prioritized | | | | | |
|  | CRS panel | SVs including CRS genes prioritized | | | | | |
|  | Size | Large SVs prioritized | | | | | |

^a^Familial Duos are families with an affected child and an affected parent available in the 100kGP dataset; sporadic duos are families with an affected child and an unaffected parent available; sporadic trios are trio samples, with affected proband and unaffected parents; singletons are affected individuals; complex cases are families with any other composition (multiple affected and/or with relative samples instead of parents).

^b^Recurrent ClinGen CNV calls were set with VarCount < 20.

^c^M&C = Manta&Canvas dataset; L = Lumpy dataset.

1. To assess the pathogenicity of the true SV calls, databases and the literature were queried manually. From gnomAD, the constraint of candidate genes was assessed, particularly for loss of function SVs such as deletions; gnomAD SVs v2.1^15^ provided further evidence on the frequency of the SV call in the gnomAD cohort. DECIPHER was queried to identify any similar SV findings from recorded cases, from which any independent cases with similar features would advance evidence of SV pathogenicity. True SVs were also assessed for conservation within the affected region and possible affected regulatory elements, using the UCSC genome browser with additional tracks including GeneHancer Regulatory Elements and Gene Interactions and Vertebrate Multiz Alignment & Conservation (100 Species). Known disease associations, particularly with skeletal abnormalities, were assessed using OMIM and literature searches.

Finally, the precise molecular rearrangements in the *TWIST1*, *ERF* and *HOXC* cluster SV/CNV (cases 20-22) were all confirmed experimentally by PCR-based breakpoint analysis (ref. 16 and data not shown).

**References**

1. Thormann A, Halachev M, McLaren W, et al. Flexible and scalable diagnostic filtering of genomic variants using G2P with Ensembl VEP. Nat Commun*.* 2019;10:2373.

2. Smedley D, Jacobsen JO, Jager M, et al. Next-generation diagnostics and disease-gene discovery with the Exomiser. Nat Protoc*.* 2015;10:2004-2015.

3. Rimmer A, 2014. https://github.com/andyrimmer/Platypus/commits/master/extensions/DeNovo/bayesianDeNovoFilter.py. Accessed 9 April 2021.

4. McLaren W, Gil L, Hunt SE, et al. The Ensembl Variant Effect Predictor. Genome Biol*.* 2016;17:122.

5. Tolchin D, Yeager JP, Prasad P, et al. De novo SOX6 variants cause a neurodevelopmental syndrome associated with ADHD, craniosynostosis, and osteochondromas. Am J Hum Genet*.* 2020;106:830-845.

6. Whiffin N, Minikel E, Walsh R, et al. Using high-resolution variant frequencies to empower clinical genome interpretation. Genet Med*.* 2017;19:1151-1158.

7. Calpena E, Cuellar A, Bala K, et al. SMAD6 variants in craniosynostosis: genotype and phenotype evaluation. Genet Med*.* 2020;22:1498-1506.

8. MacDonald JR, Ziman R, Yuen RKC, Feuk L, Scherer SW. The Database of Genomic Variants: a curated collection of structural variation in the human genome. Nucleic Acids Res*.* 2014;42:D986-D992.

9. Rehm HL, Berg JS, Brooks LD, et al. ClinGen - The Clinical Genome Resource. New Engl J Med*.* 2015;372:2235-2242.

10. UCSC Genome Browser, Schema for Gap – Gap locations, https://genome.ucsc.edu/cgi-bin/hgTables?db=hg38&hgta_group=map&hgta_track=gap&hgta_table=gap&hgta_doSchema=describe+table+schema. Accessed 9 April 2021.

11. UCSC Genome Browser, Schema for GENCODE V36, https://genome.ucsc.edu/cgi-bin/hgTables?db=hg38&hgta_group=genes&hgta_track=knownGene&hgta_table=knownGene&hgta_doSchema=describe+table+schema. Accessed 9 April 2021.

12. Genomics England, 2020. Craniosynostosis (Version 2.2). https://panelapp.genomicsengland.co.uk/panels/168/. Accessed 9 April 2021.

13. Robinson JT, Thorvaldsdottir H, Winckler W, et al. Integrative Genomics Viewer. Nat Biotechnol*.* 2011;29:24-26.

14. Belyeu, J. R., Chowdhury, M., Brown, J., et al. (2021). Samplot: a platform for structural variant visual validation and automated filtering. Genome Biol. 2021;22:161

15. Collins RL, Brand H, Karczewski KJ, et al. A structural variation reference for medical and population genetics. Nature*.* 2020;581:444-451.

16. Calpena E, McGowan SJ, Blanco Kelly F, et al. Dissection of contiguous gene effects for deletions around *ERF* on chromosome 19. Hum Mutat. 2021; doi: 10.1002/humu.24213.

**Box S1** Eligibility criteria required for inclusion of patients with CRS in 100kGP.^a^

*Inclusion criteria*:

1. Any combination of more than one major cranial vault suture fused at original presentation (from metopic, sagittal, left coronal, right coronal, left lambdoid, right lambdoid)

OR

2. Single suture synostosis accompanied by either (a) dysmorphic features or at least one major extracranial abnormality; (b) significant learning disability; (c) first or second degree relative with craniosynostosis, or offspring of consanguineous union.

*Exclusion criteria*:

1. Evidence of likely secondary cause. This is most likely to comprise (a) extreme prematurity

| <28 weeks’ gestation; (b) complications of severe perinatal asphyxia; (c) teratogenic exposure, most commonly sodium valproate; (d) compelling history of intrauterine growth restriction; (e) documented rickets (genetic or acquired). |
| --- |

2. Previously identified monogenic or chromosomal cause. Testing of the following genes should be carried out PRIOR TO RECRUITMENT where this is in line with current local practice: (i) Genome-wide copy number variation testing (e.g. aCGH, SNP array or other genomic microarray)

(ii) DNA sequencing of FGFR3-P250R, *FGFR2* exons IIIa (8) and IIIc (10), *TWIST1* exon 1 in all cases. Additional tests are available for other genes as clinically indicated (nationally commissioned testing). These tests will most commonly include the *EFNB1*, *ERF* and *TCF12* genes (mutations in each of these genes contribute ≥1% of craniosynostosis overall), and multiplex ligation-dependent probe amplification (MLPA) of *TWIST1*. Additional testing is currently offered for *FGFR1*, *FGFR2* (extended screen), *IL11RA*, *POR*, *RAB23* and *ZIC1*.

^a^Adapted from <https://www.genomicsengland.co.uk/wp-content/uploads/2018/06/Rare-Disease-Eligibility-Criteria-v1.9.0-PAR-GUI-058_approved-version-1.pdf>

**
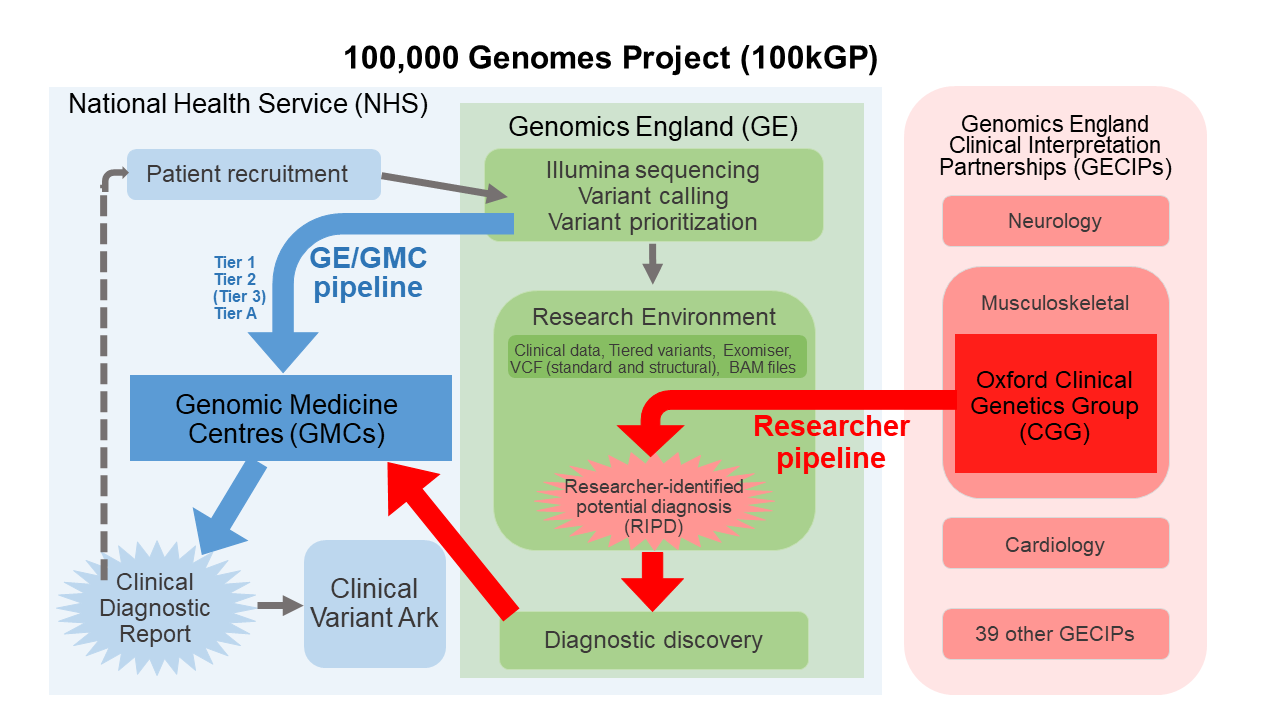
Figure S1** Relationship between the GE/GMC and Researcher pipelines for clinical diagnosis.

The GE/GMC pipeline is indicated by thick blue arrows. By comparison in the Researcher pipeline (thick red arrows), variants of potential clinical significance identified in the Research Environment are reported to the Diagnostic Discovery arm of GE using the RIPD form. Background pale blue box indicates activities taking place within the NHS (including all GE activities; green box). Pink box indicates the community of external researchers, interacting with the GE Research Environment through different GECIPs.

**Table S1** Composition of the 100kGP CRS cohort.

| **Family history** | **Analysis strategy** | **Number of families** | **Number of samples** | **Number of samples from affected individuals** | **Number of families with diagnosis** | **% families with diagnosis** |
| --- | --- | --- | --- | --- | --- | --- |
| **Sporadic** | **Singleton** | 11 | 11 | 11 | 5 | 45.5 |
|  | **Duo with unaffected parent** | 13 | 26 | 13 | 1 | 7.7 |
|  | **Trio with unaffected parents** | 72 | 224 | 72 | 24 | 33.3 |
|  | **Other** | 3 | 8 | 3 | 1 | 33.3 |
| **Familial** | **Duo or trio with affected parent** | 7 | 15 | 14 | 2 | 28.6 |
|  | **Quartet with affected sibling** | 6 | 25 | 12 | 1 | 16.7 |
|  | **Other** | 2 | 5 | 3 | 0 | 0 |
| **Total** |  | **114** | **314** | **128** | **34** | **29.8** |

**Table S2** Patterns of suture fusions in the 100kGP CRS cohort.

| **Non-syndromic or syndromic** | **Sutures fused** | **Proband** | **Affected relative** | **Total** | **Number of probands with diagnosis** | **% families with diagnosis** |
| --- | --- | --- | --- | --- | --- | --- |
| **Non-syndromic^a^** | Sagittal | (5) | (4) | (9) | 0 | 0 |
|  | Metopic | 3(1) |  | 3(1) | 1 | 25 |
|  | Unicoronal | 1 | (1) | 1(1) | 0 | 0 |
|  | Bicoronal | 5 |  | 5 | 0 | 0 |
|  | Uni- or bilambdoid | (1) | (1) | (2) | 0 | 0 |
|  | Other multiple suture | 11(1) |  | 11(1) | (1) | 8.3 |
|  | Not specified | 3(1) | (2) | 3(3) | 0 | 0 |
|  | **Total** | **23(9)** | **(8)** | **23(17)** | **1(1)** | **6.25** |
| **Syndromic** | Sagittal | 13 | 2 | 15 | 6 | 46 |
|  | Metopic | 9 | 2 | 11 | 2 | 22 |
|  | Unicoronal | 16 | 1 | 17 | 4 | 25 |
|  | Bicoronal | 6 |  | 6 | 2 | 33 |
|  | Uni- or bilambdoid | 2 |  | 2 | 1 | 50 |
|  | Other multiple suture | 29 |  | 29 | 13 | 45 |
|  | Not specified | 7 | 1 | 8 | 4 | 57 |
|  | **Total** | **82** | **6** | **88** | **32** | **39.0** |
| **Total** |  | **105(9)** | **6(8)** | **111(17)** | **33(1)** | **29.8** |

^a^Numbers in brackets refer to individuals who were classified clinically as non-syndromic, but were from pedigrees comprising two or more individuals with CRS.

**Table S5** Case reports describing CRS associated with mutations in genes identified in the RIPD cohort.

| Gene | Cases with craniosynostosis | Type (if known)^a^ | Reference |
| --- | --- | --- | --- |
| *ARID1B* | 1 | N/A | Mignot et al., 2016 |
| *CDK13* | 2 | N/A | Bostwick et al., 2017 |
|  | 1 | N/A | Tonne et al., 2021 |
| *FBXO11* | 2 | 1 sagittal, 1 metopic | Gregor et al., 2018 |
| *HNRNPK* | 1 | 1 sagittal+lambdoid | Au et al., 2015 |
|  | 1 | 1 sagittal | Lange et al., 2016 |
|  | 2 | 2 sagittal | Au et al., 2017 |

^a^N/A, not available.

Au, P. Y. B., Goedhart, C., Ferguson, M., et al. Phenotypic spectrum of Au-Kline syndrome: a report of six new cases and review of the literature. Eur J Hum Genet. 2017;26:1272-1281.

Au, P. Y. B., You, J., Caluseriu, O., et al. GeneMatcher aids in the identification of a new malformation syndrome with intellectual disability, unique facial dysmorphisms, and skeletal and connective tissue abnormalities caused by de novo variants in HNRNPK. Hum Mutat. 2015;36:1009-1014.

Bostwick, B. L., McLean, S., Posey, J. E., et al. Phenotypic and molecular characterisation of CDK13-related congenital heart defects, dysmorphic facial features and intellectual developmental disorders. Genome Med. 2017;9:73.

Gregor, A., Sadleir, L. G., Asadollahi, R., et al. De novo variants in the F-box protein FBXO11 in 20 individuals with a variable neurodevelopmental disorder. Am J Hum Genet. 2018;103:305-316.

Lange, L., Pagnamenta, A. T., Lise, S., et al. A de novo frameshift in HNRNPK causing a Kabuki-like syndrome with nodular heterotopia. Clin Genet. 2016;90:258-262.

Mignot, C., Moutard, M. L., Rastetter, A., et al. ARID1B mutations are the major genetic cause of corpus callosum anomalies in patients with intellectual disability. Brain. 2016;139:e64.

Tonne, E., Due-Tonnessen, B. J., Mero, I. L., et al. Benefits of clinical criteria and high-throughput sequencing for diagnosing children with syndromic craniosynostosis. Eur J Hum Genet. 2021;29:920-929.
